# Supplementary figures and images for: Spatial Proteomics by Parallel Accumulation‐Serial Fragmentation Supported MALDI MS/MS Imaging: A First Glance Into Multiplexed and Spatial Peptide Identification
Source: Rapid Commun Mass Spectrom. 2025 Feb 5;39(9):e10006. doi: 10.1002/rcm.10006 (PMC11799399; doi:10.1002/rcm.10006)

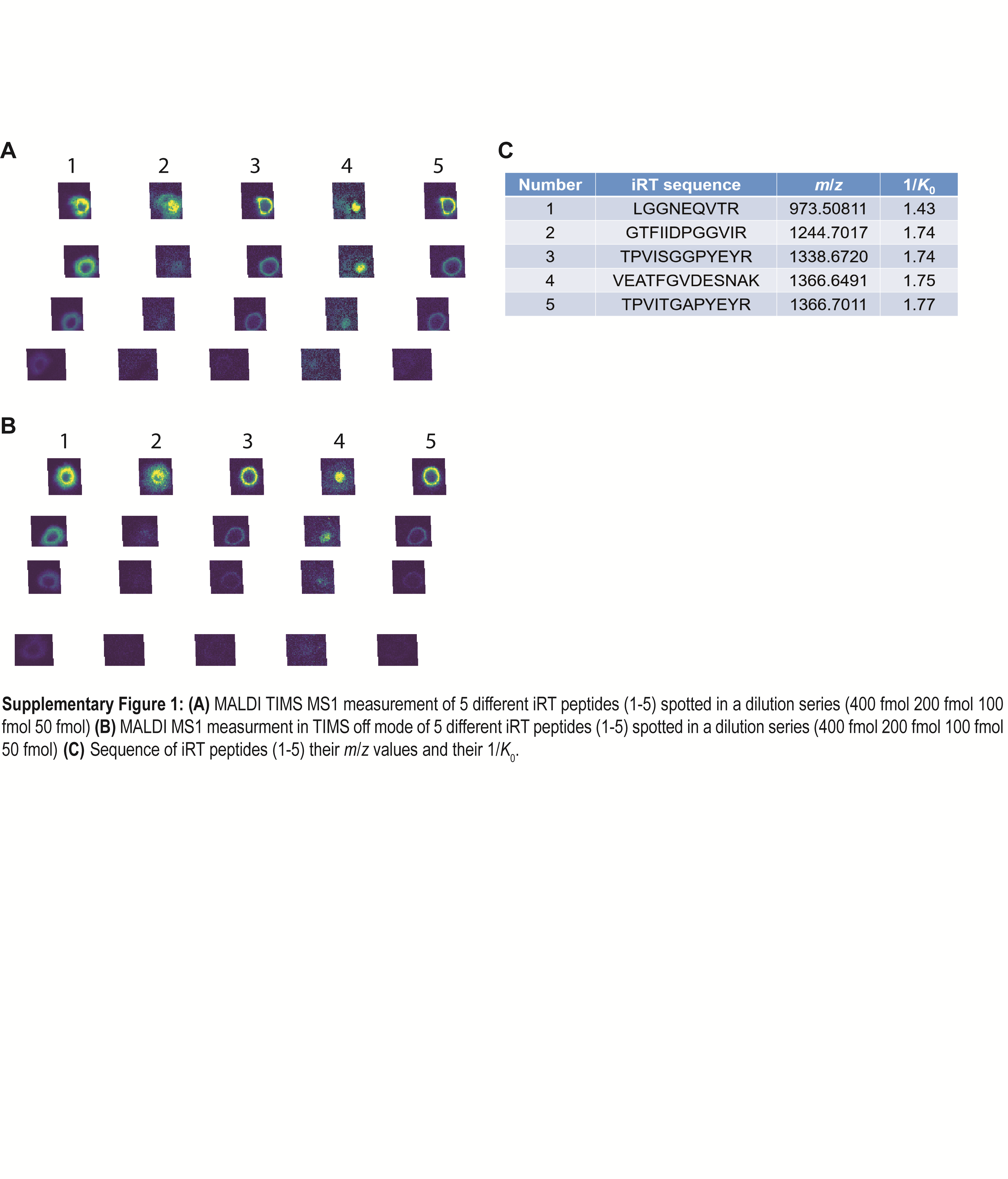

Supplement: Supplementary file 1 — Figure S1. (A) MALDI TIMS MS1 measurement of five different iRT peptides (1–5) spotted in a dilution series (400, 200, 100, and 50 fmol). (B) MALDI MS1 measurement in TIMS off mode of five different iRT peptides (1–5) spotted in a dilution series (400, 200, 100, and 50 fmol). (C) Sequence of iRT peptides (1–5) and their m/z values and 1/K 0. [file RCM-39-e10006-s002.png]

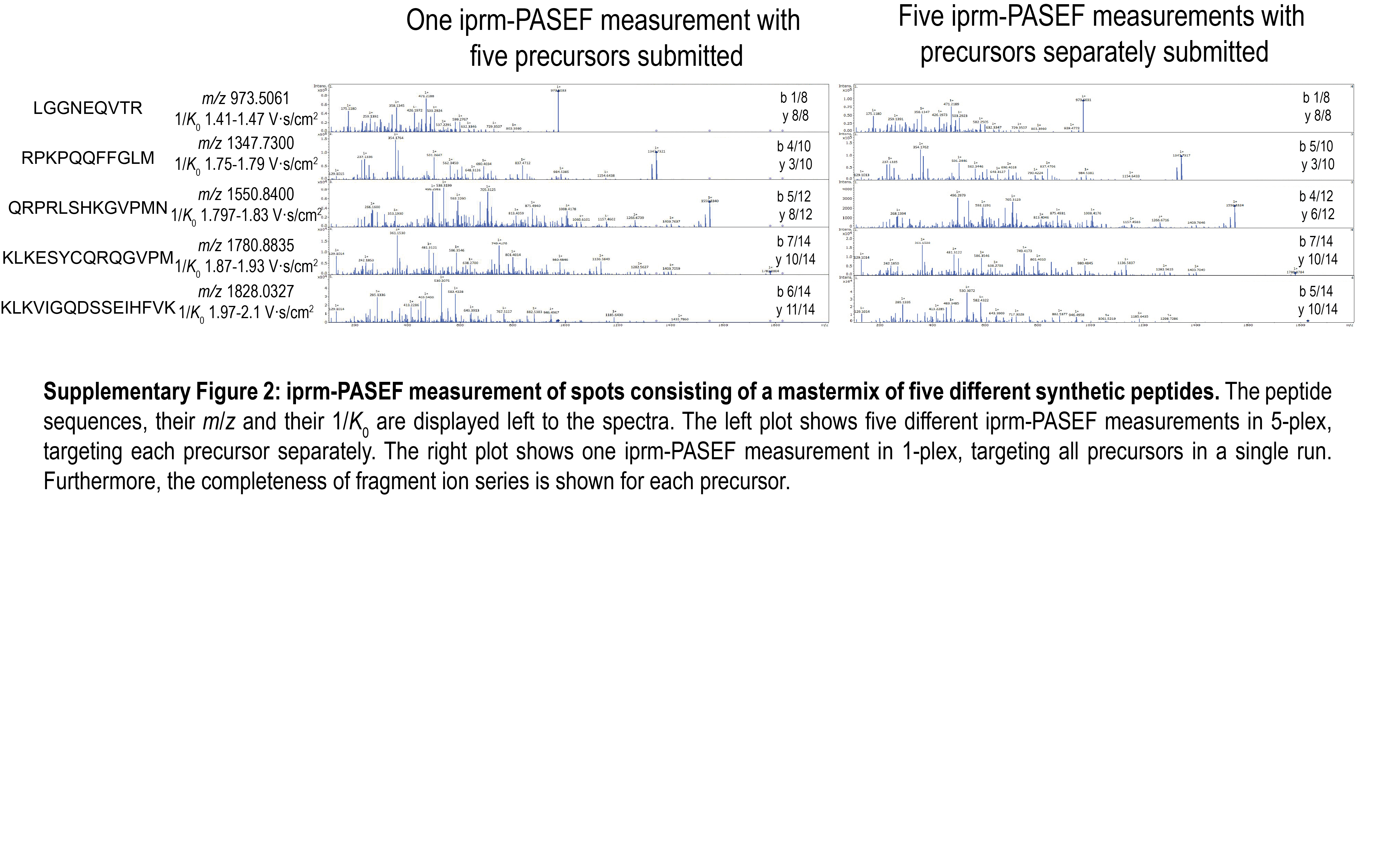

Supplement: Supplementary file 2 — Figure S2. iprm‐PASEF measurement of spots consisting of a MasterMix of five different synthetic peptides. The peptide sequences, their m/z, and their 1/K 0 are displayed left to the spectra. The left plot shows five different iprm‐PASEF measurements in five‐plex, targeting each precursor separately. The right plot shows one iprm‐PASEF measurement in one‐plex, targeting all precursors in a single run. Furthermore, the completeness of fragment ion series is shown for each precursor. [file RCM-39-e10006-s004.png]

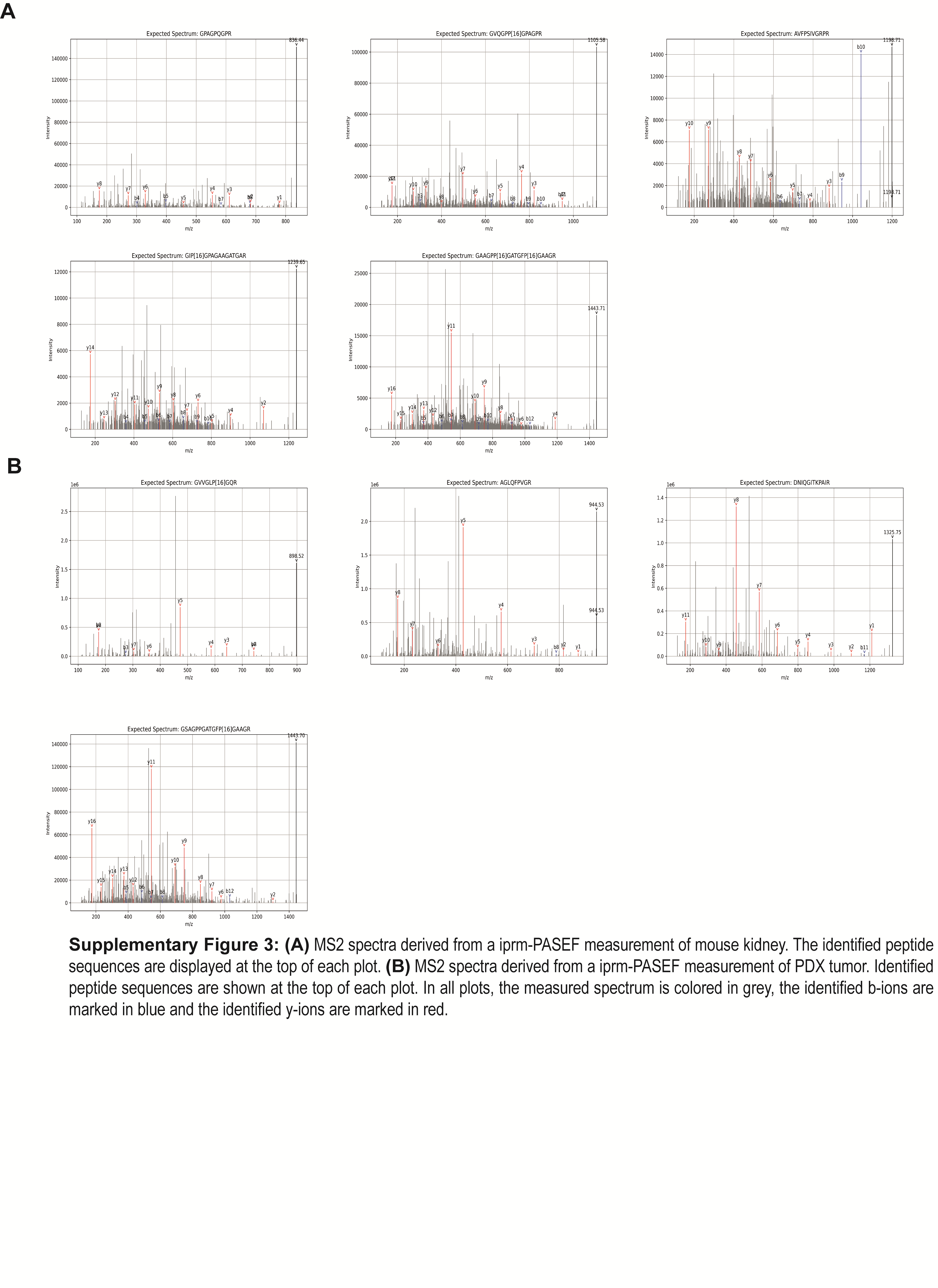

Supplement: Supplementary file 3 — Figure S3. (A) MS2 spectra derived from an iprm‐PASEF measurement of mouse kidney. The identified peptide sequences are displayed at the top of each plot. (B) MS2 spectra derived from an iprm‐PASEF measurement of PDX tumor. The identified peptide sequences are displayed at the top of each plot. In plots, the measured spectrum is colored in gray, the identified b‐ions are marked in blue, and the identified y‐ions are marked in red. [file RCM-39-e10006-s003.png]

## Slide 1
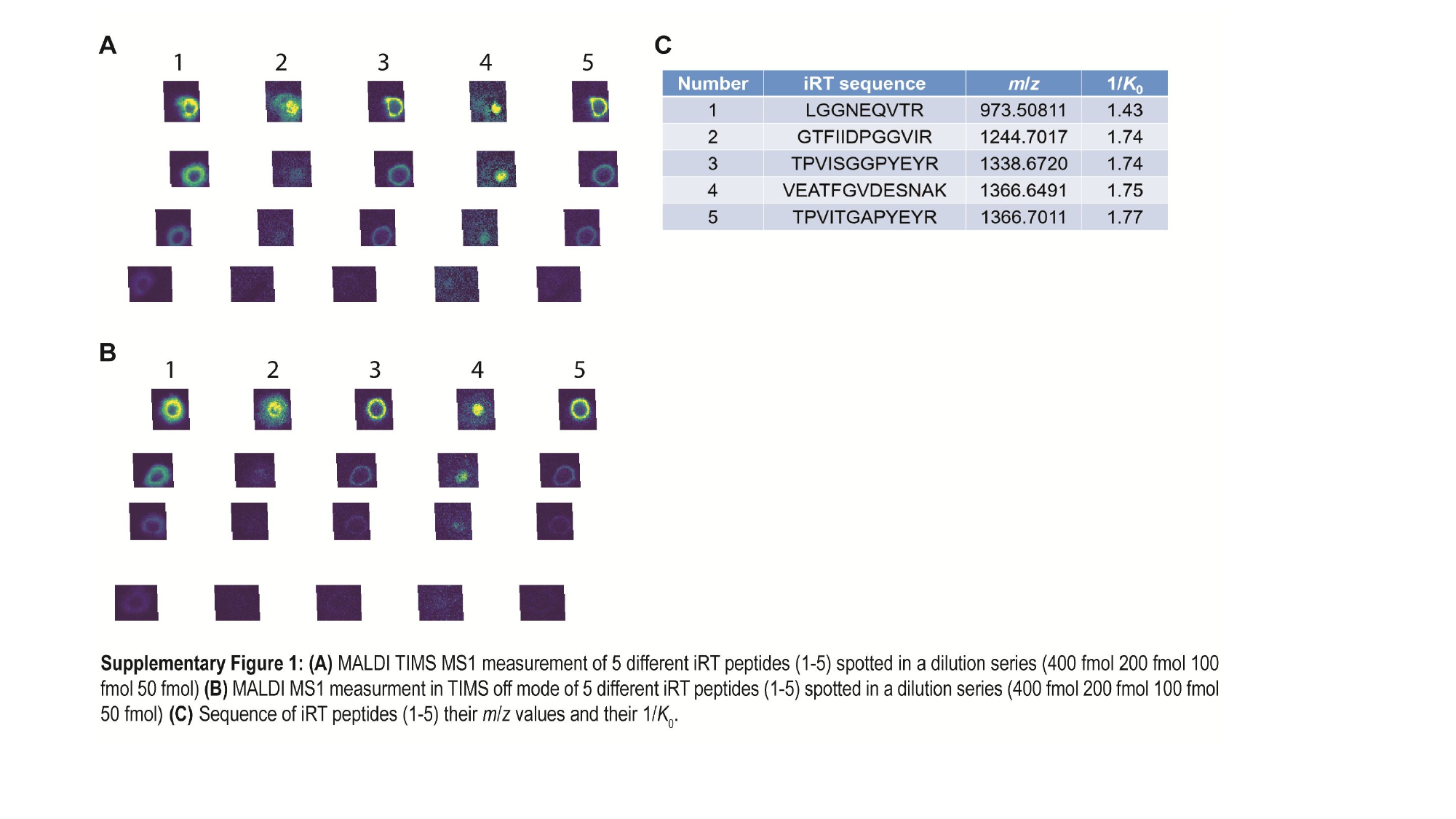

## Slide 2
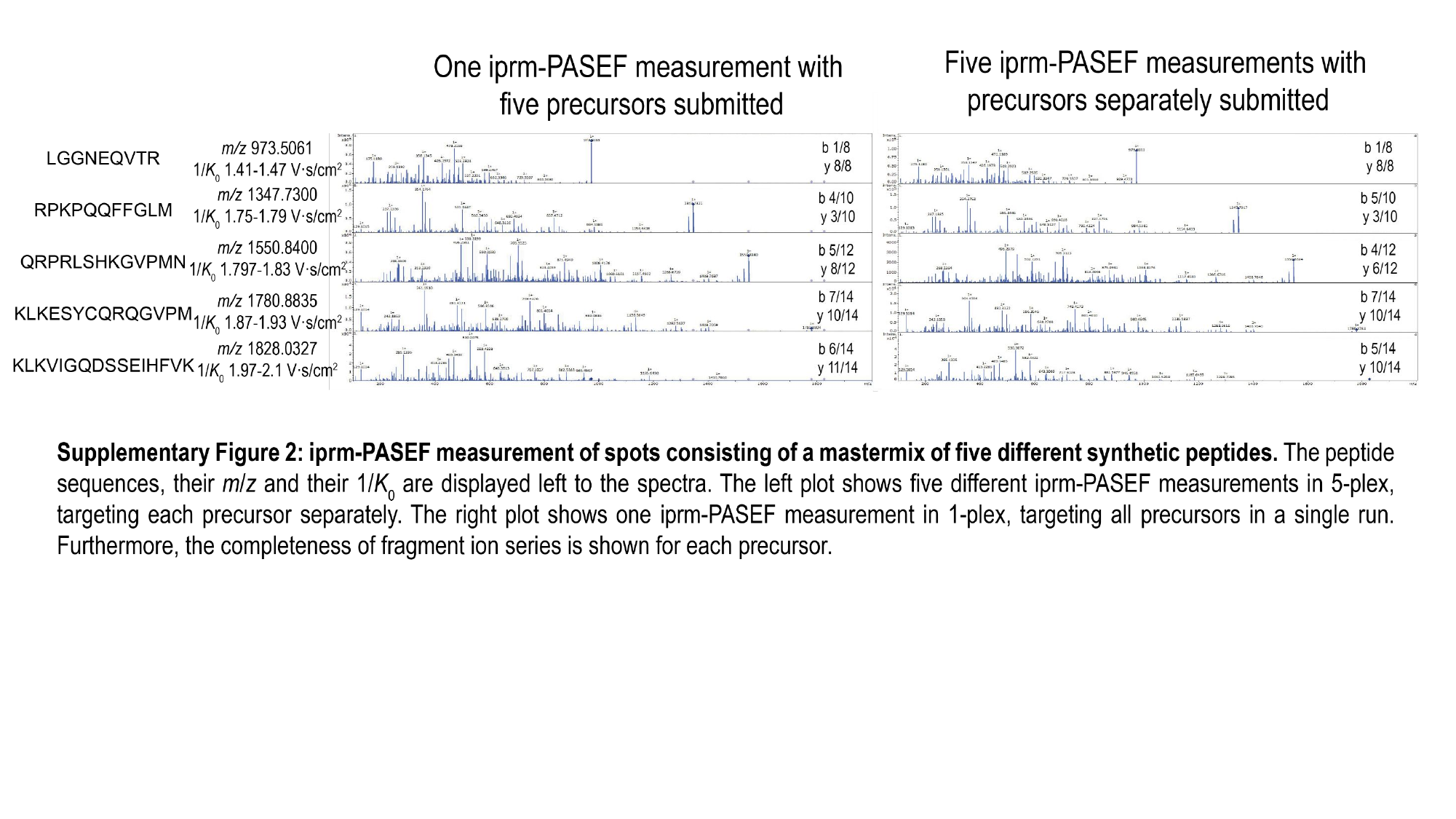

## Slide 3
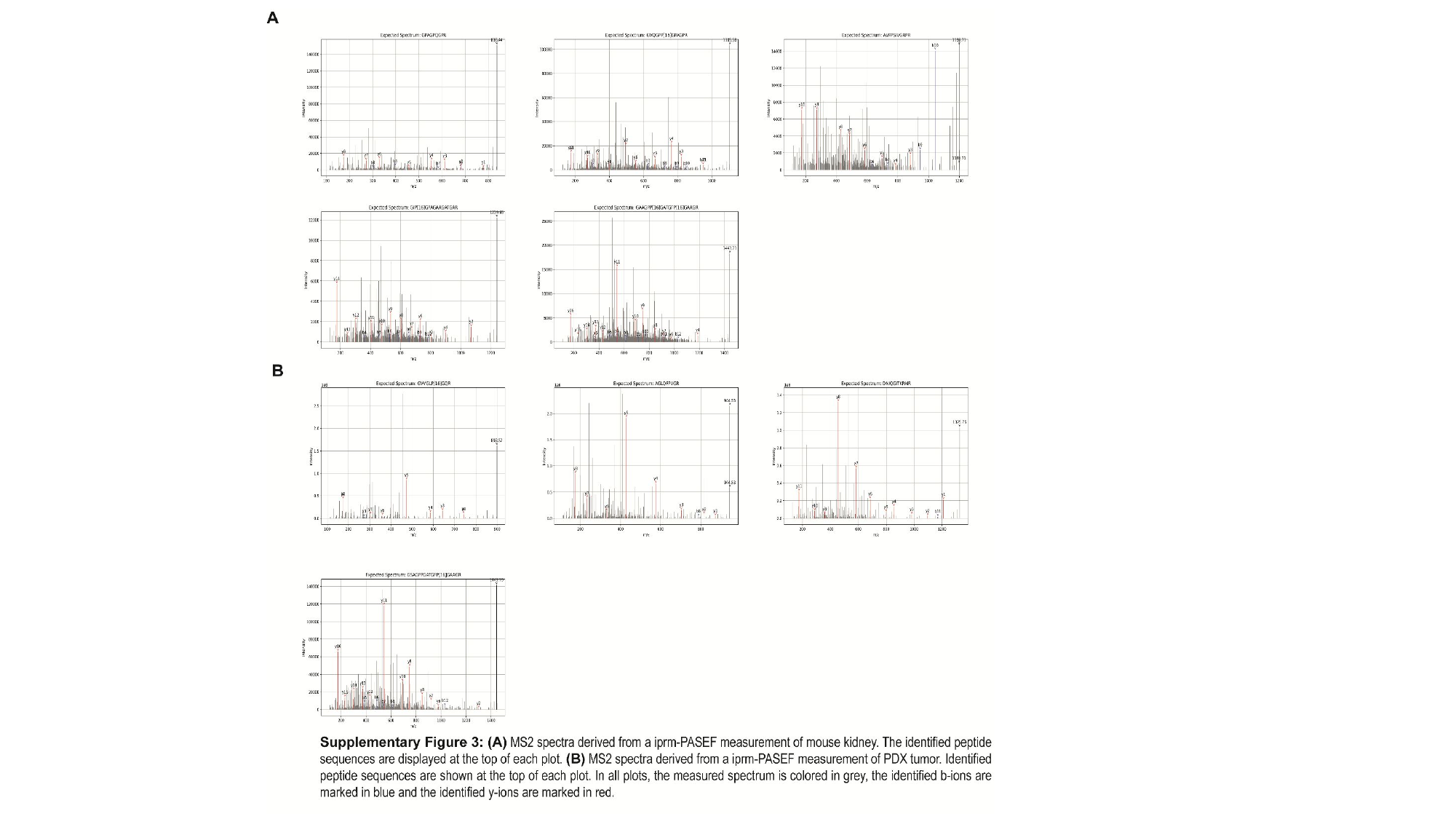

Supplement: Supplementary file 4 — Data S1 Supporting Information. [file RCM-39-e10006-s005.pptx]
