# Supplementary material for: Spatial Proteomics by Parallel Accumulation‐Serial Fragmentation Supported MALDI MS/MS Imaging: A First Glance Into Multiplexed and Spatial Peptide Identification
Source: Rapid Commun Mass Spectrom. 2025 Feb 5;39(9):e10006. doi: 10.1002/rcm.10006 (PMC11799399; doi:10.1002/rcm.10006)
Supplement: Supplementary file 5 — Table S1 Precursors selected for testing effects of including TIMS dimension on MS/MS level. Table S2: Number of b‐ and y‐ions detected in iprm‐PASEF measurements of five synthetic peptides. A MasterMix of five synthetic peptides was spotted onto an ITO slide. Precursors were either targeted one after another in an iprm‐PASEF one‐plex setup or all together in an iprm‐PASEF five‐plex setup. The fragmentation pattern including series of detected b‐ and y‐ions for each of the targeted peptides is shown in this table. Table S3: Optimization of the LC‐ESI‐TIMS‐MS/MS measurement focused on singly charged peptides. 100 ng HeLa digests were measured in three different measurement settings for optimization of 1+ peptide detection. Number of +1 peptide identifications with “Match‐type” = MS/MS. Table S4: Co‐localization scores for statistical analysis. Overview of features chosen for the final precursor list with a significant co‐localization scores (CS) determined by the “Find Values Co‐Localized to Region” tool in SCiLS Lab. For m/z 944.5373, 1325.7528, 1443.698, 836.4425, 1198.7003, 1105.5731, and 1443.6943, literature was found providing putative peptide identifications and citations are included in the table. Table S5: All precursor lists that were submitted to iprm‐PASEF analysis. Table S6: All precursors targeted with iprm‐PASEF including the detected b‐ and y‐ions calculated using the pyteomics package in python. Table S7: Co‐localization scores from fragment ions for corroboration of iprm‐PASEF peptide identification. Generated using “Find Values Co‐Localized to Feature” tool in SCiLS Lab. [file RCM-39-e10006-s001.docx]

# Supplementary Tables

**Supplementary Table 1**: Precursors selected for testing effects of including TIMS dimension on MS/MS level.

| ***m*/*z*** | **1/*K*_0_ (V•s/cm^2^)** | **Peptide_genename** |
| --- | --- | --- |
| 944.53 | 1.472 | AGLQFPVGR_H2A |
| 1,198.70 | 1.675 | AVFPSIVGRPR_ACTA |
| 1,325.75 | 1.750 | DNIQGITKPAIR_H4 |

**Supplementary Table 2: Number of b- and y-ions detected in iprm-PASEF measurements of 5 synthetic peptides.** A mastermix of 5 synthetic peptides was spotted onto an ITO slide. Precursors were either targeted one after another in an iprm-PASEF 1-plex setup or all together in an iprm-PASEF 5-plex setup. The fragmentation pattern including series of detected b- and y-ions for each of the targeted peptides is shown in this table.

| **Peptide sequence** | ***m/z* MALDI** | **1/*K*_0_ start** | **1/*K*_0_ end** | **Number of fragment ions detected** | **Detected b-ions** | **Detected y-ions** | **Experiment** |
| --- | --- | --- | --- | --- | --- | --- | --- |
| LGGNEQVTR | 973.5061 | 1.41 | 1.47 | 1/8 (b) 8/8 (y) | *m/z* (b₃)  246.18 | *m/z* (y_1_) 860.42  *m/z* (y₂) 803.40  *m/z* (y₃) 746.38  *m/z* (y₄) 632.33  *m/z* (y₅) 503.29  *m/z* (y₆) 375.23  *m/z* (y₇) 276.17  *m/z* (y₈) 175.12 | 1-plex iprm-PASEF |
| RPKPQQFFGLM | 1347.73 | 1.75 | 1.79 | 4/10 (b) 3/10 (y) | *m/z* (b_1_)  175.12  *m/z* (b_3_)  400.27  *m/z* (b_4_)  497.32  *m/z* (b₆)  753.43 | *m/z* (y₆) 614.26  *m/z* (y₇) 467.19  *m/z* (y_11_) 320.21 | 1-plex iprm-PASEF |
| QRPRLSHKGPMPF | 1550.84 | 1.797 | 1.83 | 4/12 (b) 6/12 (y) | *m/z* (b_4_)  556.33  *m/z* (b_5_)  669.42  *m/z* (b_6_)  756.45  *m/z* (b_12_)  1403.76 | *m/z* (y₂) 1266.67  *m/z* (y₅) 900.43  *m/z* (y₆) 813.40  *m/z* (y₇) 676.35  *m/z* (y₈) 548.25  *m/z* (y_11_) 263.14 | 1-plex iprm-PASEF |
| KLKESYCQRQGVPMN | 1780.8835 | 1.87 | 1.93 | 7/14 (b) 10/14 (y) | *m/z* (b_3_)  388.23  *m/z* (b_4_)  517.33  *m/z* (b_5_)  604.30  *m/z* (b_6_)  767.43  *m/z* (b_8_)  998.42  *m/z* (b_10_)  1282.56  *m/z* (b_14_)  1666.84 | *m/z* (y_4_) 1282.56  *m/z* (y_5_) 1195.53  *m/z* (y_6_) 1032.47  *m/z* (y_7_) 929.46  *m/z* (y_9_) 801.40  *m/z* (y_9_) 645.30  *m/z* (y_10_) 517.19  *m/z* (y_11_) 460.22  *m/z* (y_12_) 361.15  *m/z* (y_13_) 264.10 | 1-plex iprm-PASEF |
| KLKVIGQDSSEIHFKV | 1828.0327 | 1.97 | 2.1 | 6/15 (b) 10/15 (y) | *m/z* (b_3_)  388.23  *m/z* (b_5_)  600.30  *m/z* (b_6_)  657.44  *m/z* (b7)  785.52  *m/z* (b_8_)  900.55  *m/z* (b_9_)  987.58 | *m/z* (y_4_) 1359.68  *m/z* (y_5_) 1246.60  *m/z* (y_7_) 1061.52  *m/z* (y_8_) 946.50  *m/z* (y_9_) 859.46  *m/z* (y_10_) 772.43  *m/z* (y_11_) 643.39  *m/z* (y_12_) 530.31  *m/z* (y_13_) 393.25  *m/z* (y_14_) 246.18 | 1-plex iprm-PASEF |
| LGGNEQVTR | 973.5061 | 1.41 | 1.47 | 1/8 (b) 8/8 (y) | *m/z* (b₃)  246.18 | *m/z* (y_1_) 860.42  *m/z* (y₂) 803.40  *m/z* (y₃) 746.38  *m/z* (y₄) 632.33  *m/z* (y₅) 503.29  *m/z* (y₆) 375.23  *m/z* (y₇) 276.17  *m/z* (y₈) 175.12 | 5-plex iprm-PASEF |
| RPKPQQFFGLM | 1347.73 | 1.75 | 1.79 | 5/10 (b) 3/10 (y) | *m/z* (b_1_)  175.12  *m/z* (b_3_)  400.27  *m/z* (b_4_)  497.32  *m/z* (b₆)  753.43  *m/z* (b_7_)  900.49 | *m/z* (y₆) 614.26  *m/z* (y₇) 467.19  *m/z* (y₈) 320.21 | 5-plex iprm-PASEF |
| QRPRLSHKGPMPF | 1550.84 | 1.797 | 1.83 | 5/12 (b) 8/12 (y) | *m/z* (b_3_)  400.23  *m/z* (b_4_)  556.33  *m/z* (b_5_)  669.42  *m/z* (b_6_)  756.45  *m/z* (b_12_)  1403.76 | *m/z* (y₂) 1266.67  *m/z* (y₄) 1013.52  *m/z* (y₅) 900.43  *m/z* (y₆) 813.40  *m/z* (y₇) 676.35  *m/z* (y₈) 548.25  *m/z* (y_9_) 491.23  *m/z* (y_11_) 263.14 | 5-plex iprm-PASEF |
| KLKESYCQRQGVPMN | 1780.8835 | 1.87 | 1.93 | 7/14 (b) 10/14 (y) | *m/z* (b_3_)  388.23  *m/z* (b_4_)  517.33  *m/z* (b_5_)  604.30  *m/z* (b_6_)  767.43  *m/z* (b_8_)  998.42  *m/z* (b_10_)  1282.56  *m/z* (b_14_)  1666.84 | *m/z* (y_4_) 1282.56  *m/z* (y_5_) 1195.53  *m/z* (y_6_) 1032.47  *m/z* (y_7_) 929.46  *m/z* (y_8_) 801.40  *m/z* (y_9_) 645.30  *m/z* (y_10_) 517.19  *m/z* (y_11_) 460.22  *m/z* (y_12_) 361.15  *m/z* (y_13_) 264.10 | 5-plex iprm-PASEF |
| KLKVIGQDSSEIHFKV | 1828.0327 | 1.97 | 2.1 | 7/15 (b) 11/15 (y) | *m/z* (b_3_)  388.23  *m/z* (b_4_)  487.21  *m/z* (b_5_)  600.30  *m/z* (b_6_)  657.44  *m/z* (b_7_)  785.52  *m/z* (b_8_)  900.55  *m/z* (b_9_)  987.58 | *m/z* (y_4_) 1359.68  *m/z* (y_5_) 1246.60  *m/z* (y_6_) 1189.58  *m/z* (y_7_) 1061.52  *m/z* (y_8_) 946.50  *m/z* (y_9_) 859.46  *m/z* (y_10_) 772.43  *m/z* (y_11_) 643.39  *m/z* (y_12_) 530.31  *m/z* (y_13_) 393.25  *m/z* (y_14_) 246.18 | 5-plex iprm-PASEF |

**Supplementary Table 3**: Optimization of the LC-ESI-TIMS-MS/MS measurement focused on singly charged peptides. 100 ng HeLa digests were measured in three different measurement settings for optimization of 1+ peptide detection. Number of +1 peptide identifications with “Match-type”= MS/MS.

|  | **Prepulse storage** | **Transfer time** | **Def Delta 1** | **Funnel 1 RF** | **Funnel 2 RF** | **Collision energy** | **Collision RF** | **Ion Energy** | **1/*K*_0_ range** | **TIMS in pressure** | **+1 Peptide ID** |
| --- | --- | --- | --- | --- | --- | --- | --- | --- | --- | --- | --- |
| DDAPasef Proteomics | 12 µs | 60 µs | 70 V | 450 Vpp | 200 Vpp | 20-59 eV | 1,500 Vpp | 5eV | 0.6-1.6 V*s/cm^2^ | 2.4 mbar | 0 |
| DDAPasef 1+ | 15 µs | 120 µs | 70 V | 450 Vpp | 200 Vpp | 30-74 eV | 2,000 Vpp | 5eV | 0.7-1.75 V*s/cm^2^ | 2.4 mbar | 2,359 |
| DDAPasef 1+ | 15 µs | 120 µs | 70 V | 450 Vpp | 200 Vpp | 30-74 eV | 2,000 Vpp | 5eV | 0.7-2.1 V*s/cm^2^ | 2 mbar | 3,211 |
| DDAPasef 1+ opt | 15 µs | 70 µs | 80 V | 250 Vpp | 500 Vpp | 20-75 eV | 2,500 Vpp | 5eV | 0.7-2.1 V*s/cm^2^ | 2 mbar | 3,646 |

**Supplementary Table 4: Co-localization scores for statistical analysis.** Overview of features chosen for the final precursor list with a significant co-localization scores (CS) determined by the “Find Values Co-Localized to Region” tool in SCiLS Lab. For *m*/*z* 944.5373, 1,325.7528, 1,443.698, 836.4425, 1,198.7003, 1,105.5731 and 1,443.6943, literature was found providing putative peptide identifications and citations are included in the table.

| ***m*/*z*** | **1/*K*_0_  (V•s/cm^2^)** | **CS** | **Tissue** | **Literature** |
| --- | --- | --- | --- | --- |
| 898.5029 | 1.4247 | 0.74 | PDX tumor | - |
| 944.5373 | 1.4714 | -0.71 | PDX tumor | PMID: 27061135 |
| 1,325.7528 | 1.77 | -0.77 | PDX tumor | PMID: 34206844, 31664609,34572274,38492056,18712763,38928454 |
| 1,443.698 | 1.7822 | 0.76 | PDX tumor | PMID: 26505774 |
| 836.4425 | 1.32 | 0.79 | mouse kidney | PMID: 27696080, 30548962, 36768889, 34572274, 31664609, 27939604 |
| 1,198.7003 | 1.69 | -0.15 | mouse kidney | PMID: 35684402, 27939604 |
| 1,105.5731 | 1.55 | 0.74 | mouse kidney | PMID: 27061135 |
| 1,239.6418 | 1.71 | 0.71 | mouse kidney | - |
| 1,443.6943 | 1.74 | 0.75 | mouse kidney | PMID: 26505774 |

**Supplementary Table 5:** All precursor lists that were submitted to iprm-PASEF analysis.

**A:** Mouse kidney precursor list for iprm-PASEF. Precursors 1260.61 *m*/*z* and 852.43 *m*/*z* were included because of ion mobility window availability but could not be identified by MASCOT.

| ***m*/*z*** | **1/*K*_0_ start** | **1/*K*_0_ end** |
| --- | --- | --- |
| 836.43 | 1.31 | 1.35 |
| 852.43 | 1.27 | 1.3 |
| 1,106.58 | 1.44 | 1.48 |
| 1,199.7 | 1.68 | 1.7 |
| 1,239.64 | 1.701 | 1.73 |
| 1,260.61 | 1.64 | 1.67 |
| 1,443.6819 | 1.7301 | 1.78 |

**B:** PDX tumor precursor list for iprm-PASEF

| ***m*/*z*** | **1/*K*_0_ start** | **1/*K*_0_ end** |
| --- | --- | --- |
| 898.5 | 1.4 | 1.43 |
| 944.5 | 1.441 | 1.49 |
| 1,325.72 | 1.75 | 1.775 |
| 1,443.67 | 1.781 | 1.81 |

**Supplementary Table 6:** All precursors targeted with iprm-PASEF including the detected b- and y-ions calculated using the pyteomics package in python.

| **Peptide sequence** | ***m/z* MALDI** | **Detected b-ions** | **Detected y-ions** | **Measured tissue** |
| --- | --- | --- | --- | --- |
| GVVGLP[16]GQR | 898.5029 | *m/z* (b₂) 175.12  *m/z* (b₃) 274.19  *m/z* (b₈) 742.42 | *m/z* (y₂) 742.42  *m/z* (y₃) 643.35  *m/z* (y₄) 586.33  *m/z* (y₅) 473.25  *m/z* (y₆) 360.20  *m/z* (y₇) 303.18  *m/z* (y₈) 175.12 | PDX tumor |
| AGLQFPVGR | 944.5373 | *m/z* (b₈) 788.42 | *m/z* (y₁) 873.49  *m/z* (y₂) 816.47  *m/z* (y₃) 703.39  *m/z* (y₄) 575.33  *m/z* (y₅) 428.26  *m/z* (y₆) 331.21  *m/z* (y₇) 232.14  *m/z* (y₈) 175.12 | PDX tumor |
| DNIQGITKPAIR | 1325.7528 | *m/z* (b₁₁) 1169.65 | *m/z* (y₁) 1210.72  *m/z* (y₂) 1096.68  *m/z* (y₃) 983.60  *m/z* (y₄) 855.54  *m/z* (y₅) 798.52  *m/z* (y₆) 685.43  *m/z* (y₇) 584.39  *m/z* (y₈) 456.29  *m/z* (y₉) 359.24  *m/z* (y₁₀) 288.20  *m/z* (y₁₁) 175.12 | PDX tumor |
| GSAGPPGATGFP[16]GAAGR | 1443.698 | *m/z* (b₅) 388.18  *m/z* (b₆) 485.22  *m/z* (b₇) 542.26  *m/z* (b₈)613.32  *m/z* (b₁₂) 1031.47 | *m/z* (y₂) 1299.61  *m/z* (y₆) 977.48  *m/z* (y₇) 920.46  *m/z* (y₈) 849.42  *m/z* (y₉) 748.37  *m/z* (y₁₀) 691.35  *m/z* (y₁₁) 544.28  *m/z* (y₁₂) 431.23  *m/z* (y₁₃) 374.21  *m/z* (y₁₄) 303.18  *m/z* (y₁₅) 232.14  *m/z* (y₁₆) 175.12 | PDX tumor |
| GPAGPQGPR | 836.4425 | *m/z* (b₄) 301.15  *m/z* (b₅) 398.20  *m/z* (b₇) 583.28  *m/z* (b₈)680.34 | *m/z* (y₁) 779.41  *m/z* (y₂) 682.36  *m/z* (y₃) 611.33  *m/z* (y₄) 554.30  *m/z* (y₅) 457.25  *m/z* (y₆) 329.19  *m/z* (y₇) 272.17  *m/z* (y₈) 175.12 | mouse kidney |
| GVQGPP[16]GPAGPR | 1105.5731 | *m/z* (b₂) 175.12  *m/z* (b₃) 303.03  *m/z* (b₇) 627.21  *m/z* (b₈) 724.33  *m/z* (b₉) 795.30  *m/z* (b₁₀) 852.39  *m/z* (b₁₁) 949.48 | *m/z* (y₂) 949.48  *m/z* (y₃) 821.42  *m/z* (y₄) 764.40  *m/z* (y₅) 667.35  *m/z* (y₆) 554.30  *m/z* (y₇) 497.28  *m/z* (y₈) 400.23  *m/z* (y₉) 329.19  *m/z* (y₁₀) 272.17  *m/z* (y₁₁) 175.12 | mouse kidney |
| AVFPSIVGRPR | 1198.7003 | *m/z* (b₆) 633.32  *m/z* (b₇) 732.41  *m/z* (b₉) 945.55  *m/z* (b₁₀) 1042.60 | *m/z* (y₃) 881.53  *m/z* (y₄) 784.48  *m/z* (y₅) 697.45  *m/z* (y₆) 584.36  *m/z* (y₇) 485.29  *m/z* (y₈) 428.27  *m/z* (y₉) 272.17  *m/z* (y₁₀) 175.12 | mouse kidney |
| GIP[16]GPAGAAGATGAR | 1239.6418 | *m/z* (b₄) 359.13  *m/z* (b₅) 456.22  *m/z* (b₆) 527.25  *m/z* (b₇) 584.31  *m/z* (b₈) 655.38  *m/z* (b₉) 726.32  *m/z* (b₁₀) 783.36 | *m/z* (y₂) 1069.54  *m/z* (y₄) 899.47  *m/z* (y₅) 802.41  *m/z* (y₆) 731.38  *m/z* (y₇) 674.36  *m/z* (y₈) 603.32  *m/z* (y₉) 532.28  *m/z* (y₁₀) 475.26  *m/z* (y₁₁) 404.22  *m/z* (y₁₂) 303.18  *m/z* (y₁₃) 246.16  *m/z* (y₁₄) 175.12 | mouse kidney |
| GAAGPP[16]GATGFP[16]GAAGR | 1443.6943 | *m/z* (b₅) 372.07  *m/z* (b₆) 485.15  *m/z* (b₇) 542.21  *m/z* (b₈) 613.25  *m/z* (b₉) 714.31  *m/z* (b₁₀) 771.28  *m/z* (b₁₁) 918.39  *m/z* (b₁₂) 1031.36 | *m/z* (y₄) 1187.58  *m/z* (y₆) 977.48  *m/z* (y₇) 920.46  *m/z* (y₈) 849.42  *m/z* (y₉) 748.37  *m/z* (y₁₀) 691.35  *m/z* (y₁₁) 544.28  *m/z* (y₁₂) 431.23  *m/z* (y₁₃) 374.21  *m/z* (y₁₄) 303.18  *m/z* (y₁₅) 232.14  *m/z* (y₁₆) 175.12 | mouse kidney |

**Supplementary Table 7:** Co-localization scores from fragment ions for corroboration of iprm-PASEF peptide identification. Generated using “Find Values Co-Localized to Feature” tool in SCiLS Lab.

| ***m*/*z* precursor** | **Fragment ion** | **Co-localization score** | **Tissue** |
| --- | --- | --- | --- |
| 836.4425 | 611.328 (y_3_) | 0.4599 | mouse kidney |
| 836.4425 | 554.3025 (y_4_) | 0.4949 | mouse kidney |
| 836.4425 | 397.2158 (b_5_) | 0.5625 | mouse kidney |
| 836.4425 | 329.1929 (y_6_) | 0.4699 | mouse kidney |
| 836.4425 | 272.1689 (y_7_) | 0.4035 | mouse kidney |
| 1,325.7528 | 1,210.7247 (y_1_) | 0.9683 | PDX tumor |
| 1,325.7528 | 855.5296 (y_4_) | 0.7638 | PDX tumor |
| 1,325.7528 | 798.5168 (y_5_) | 0.6254 | PDX tumor |
| 1,325.7528 | 685.4353 (y_6_) | 0.5655 | PDX tumor |
| 1,325.7528 | 584.3861 (y_7_) | 0.7317 | PDX tumor |
